# In Vitro Wound Healing Potential of Flavonoid *C*-Glycosides from Oil Palm (*Elaeis guineensis* Jacq.) Leaves on 3T3 Fibroblast Cells

**DOI:** 10.3390/antiox9040326

**Published:** 2020-04-17

**Authors:** Mohamad Shazeli Che Zain, Soo Yee Lee, Murni Nazira Sarian, Sharida Fakurazi, Khozirah Shaari

**Affiliations:** 1 Laboratory of Natural Products, Institute of Bioscience, Universiti Putra Malaysia, Serdang 43400, Selangor, Malaysia; shazelizain@gmail.com (M.S.C.Z.); daphne.leesooyee@gmail.com (S.Y.L.); 2 Laboratory of Vaccine and Immunotherapeutic, Institute of Bioscience, Universiti Putra Malaysia, Serdang 43400, Selangor, Malaysia; murninazira88@gmail.com (M.N.S.); sharida@upm.edu.my (S.F.); 3 Institute of Systems Biology (INBIOSIS), Universiti Kebangsaan Malaysia UKM, Bangi 43600, Selangor, Malaysia

**Keywords:** oil palm (*Elaeis guineensis* Jacq.) leaf, crude extracts, total flavonoids, flavonoid *C*-glycosides, wound healing

## Abstract

Oil palm (*Elaeis guineensis* Jacq.) leaves (OPL) are widely available at zero cost in Southeast Asia countries, especially in Malaysia and Indonesia due to large scale oil palm plantations. OPLs contain a large amount of flavonoids in particular flavonoid *C*-glycosides, which are known to possess useful biological properties including antioxidant and wound healing properties. The present study aimed at evaluating the wound healing efficacy of OPL in various solvent extracts and flavonoid enriched fractions and to determine the contribution of flavonoid *C*-glycosides (orientin, isoorientin, vitexin and isovitexin) using *in-vitro* scratch assay on 3T3 fibroblast cells. Solvent crude extracts with different polarity were screened and the most active extract was subjected to acid hydrolysis. The crude and acid hydrolysed extracts were further enriched using macroporous resins, XAD7HP. UHPLC-UV/PDA and LC-MS/MS analysis were applied for identification and confirmation of flavonoid *C*-glycosides. The wound healing properties comprised of cell viability, cell proliferation and cell migration were studied. Allantoin was used as a positive control to compare the efficacy among the tested samples. The results revealed all OPL crude extracts, flavonoid enriched fractions and flavonoid *C*-glycosides were non-toxic at concentrations below 25 µg/mL and showed better cell proliferation and migration activities at low concentrations than higher concentrations.. This study also demonstrated orientin, isoorientin, vitexin and isovitexin presented in OPL extracts and flavonoid enriched fractions stimulated proliferation and migration of 3T3 fibroblast cells. Hence, these findings may pose potential therapeutic bioactive agents for wound healing by enhancing fibroblast proliferation and migration.

## 1. Introduction

Skin plays a vital role in protecting the human body from external harmful agents such as microorganisms, toxins, and chemicals that may disrupt the defensive barrier when injuries or wounds occur. Hence, it is necessary to restore the structural integrity of wounded skin to restore its normal function with high efficiency. Wound healing is a complex and dynamic process in regenerating and replacing injured cells at the wound sites [1], which involved series of interactions between biological and molecular events including cell migration, cell proliferation and extracellular matrix (ECM) deposition [2,3,4]. 

Owing to the superiority in healing wounds naturally, wounds on the skin may sometimes encounter a state of pathological inflammation caused by incomplete, postponed or uncoordinated healing processes that show impaired or delayed acute and chronic wounds [5]. Conventionally, these problems are solved by providing antibiotics, anti-inflammatory agents or a combination of both. However, some of the prescribed drugs containing trypsin, Balsam Peru and castor oil, collagenase and silver sulfadiazine, are not only expensive, but some sensitive patients have to endure unpleasant side effects [6] such as skin irritation, rashes, blood dyscrasias and life-threatening cutaneous reactions [7]. Henceforth, the discovery of novel therapeutic agents expected to be more affordable and effective in comparison with the available drugs is needed.

Natural products are known to be an alternative source of natural therapeutic agents in the treatment of wound healing due to their effective application as of centuries ago [8,9]. At present, there has been growing interest in oil palm (*Elaeis guineensis*) leaves (OPL) from local researchers, as OPL are not only abundantly available at zero cost as it is widely generated from the palm oil industry; yet, fascinatingly, recent studies revealed its high potential on wound healing treatment [10,11]. In separate studies, phytochemical screening revealed that flavonoids including flavonoid *C*-glycosides and flavonoid *O*-glycosides are the major compounds presented in OPL [12,13]. Compared to flavonoid *O*-glycosides, the flavonoid *C*-glycosides are less common in occurrence but still regarded as therapeutically important considering that these compounds have been shown to be have interesting biological properties including wound healing properties. The importance of flavonoid *C*-glycosides is also related to the fact that these compounds have minimal conformational differences to their *O*-glycosides and that they are resistant to enzymatic or acidic hydrolysis since the functional group of the anomeric center has been transformed from acetal to ether. However, there is scarce knowledge on their mode of action and correlation of these flavonoid *C*-glycosides on the wound healing process.

Therefore, the objective of the present study was to evaluate the efficacy of different extracts and flavonoid enriched fractions of oil palm (*Elaeis guineensis*) leaves in promoting proliferation and migration of monolayer 3T3 fibroblasts and to determine the contribution of flavonoid *C*-glycosides for wound healing potential. Solvents with differing polarity were used to prepare the various crude extracts, while macroporous resin and its combination with acid hydrolysis were employed to produce the flavonoid and flavonoid *C*-glycoside enriched fractions, respectively. In-vitro scratch assay was applied in this study as the approach has proven to be a valuable and inexpensive method to preliminarily study wound healing potential of drugs [14]. Prior to all wound healing tests, cytotoxicity test was carried out to evaluate the toxicity of the samples. The wound healing activities were compared with positive control drug, allantoin which known to possess high wound healing properties and it has been used in pharmaceutical preparation for more than 70 years [15]. Previous studies provided evidences that flavonoid *C*-glycosides including orientin, isoorientin, vitexin and isovitexin showed good anti-inflammatory activity which is one of the important phases in wound healing mechanisms [16,17]. Hence, the wound healing activities of these compounds were also evaluated in order to determine their contribution towards the wound healing potential of OPL. The findings of this study will provide insight of in-depth understanding of the correlation of flavonoid *C*-glycosides obtained from oil palm leaves on the cell proliferation and migration of fibroblasts. Hence, it could be a promising platform for discovering natural remedies to enhance the wound healing process. 

## 2. Materials and Methods 

### 2.1. Chemicals and Reagents

Dimethyl sulfoxide (DMSO), absolute methanol, n-hexane, ethyl acetate and 95% ethanol were supplied by R&M Chemicals (UK). Water was purified by a MilliQ system (Millipore, Bedford, MA, USA). UPLC grade water and acetonitrile were purchased from Merck (Darmstadt, Germany). XAD7 7HP and allantoin was supplied by Sigma (Aldrich, Germany). Vitexin (apigenin-8-*C*-glucoside) (>98.0%), isovitexin (apigenin-6-*C*-glucoside) (>98.0%), orientin (luteolin-8-*C*-glucoside) (>98.0%) and isoorientin (luteolin-8-*C*-glucoside) (>98.0%) were supplied by ChemFaces (Wuhan, China). Dulbecco’s modified eagle medium (DMEM), phosphate buffer saline (PBS) solution and thiazoyl blue tetrazolium bromide (MTT) were purchased from Solarbio (Beijing, China). Penicillin-streptomycin and trypsin were supplied by Nacalai Tesque (Kyoto, Japan). Fetal bovine serum was brought from Tico Europe (Amstelveen, Netherlands). Cell counting KIT-8 (CCK-8) was provided by Dojindo Lab (Kumamoto, Japan). 

### 2.2. Sampling

The oil palm (*Elaeis guineensis* Jacq.) mature leaves (OPL) were harvested from Universiti Agricultural Park, UPM, Malaysia. The healthy oil palm tree aged 5 years old was selected and labelled for two years’ use. The mature leaves were taken from the 17th frond, which was counted from “frond 0” in a spiral manner from the apex. For uniformity, this sampling method followed the routine sampling practice by agronomist [18]. The OPL was harvested from 8.00 am to 9.00 am to minimize variation. The OPL was authenticated by an in-house botanist of Institute of Bioscience, UPM and the voucher specimen (SK 3332/18) was deposited in the Herbarium of Institute of Bioscience, UPM. 

### 2.3. Sample Preparation

**Figure 1:**
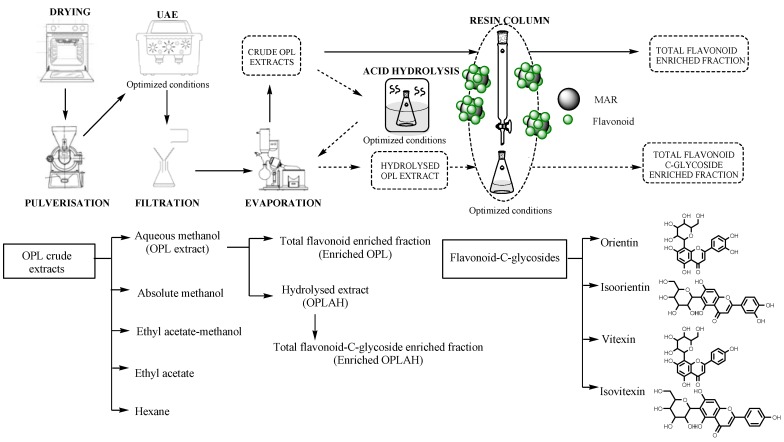
Graphical flow chart of various sample preparations. UAE = ultrasound assisted extraction, MAR = macroporous resin, solid arrow = pathway to obtain total flavonoid enriched fraction, dotted arrow= pathway to obtain total flavonoid *C*-glycosides enriched fraction.

The oil palm leaves (OPL) were cleaned using water after harvesting. The rachises of the OPL were removed. The OPL were measured and cut to approximately 2.54 cm (an inch length). The leaflets were subjected to oven drying at 35 °C by using a universal drying oven (model 100–800, Memmert, Schwabach, Germany) until constant weight was obtained. The dried OPL were pulverised using mechanical grinder (Philips, HR2056, Eindhoven, Netherlands) until powdered-size particles formed, followed by sieving with 300 µm pore size sieve. The OPL powders were kept in a labelled, air-tight container at −80 °C. To aid visualization, Figure 1 showed graphical flow chart of sample preparation. 

### 2.4. Preparation of Crude Oil Palm Leaf (OPL) Extracts

The sample preparation was performed as described previously by Tahir et al. [12], with modifications. The 0.1 g OPL powder was mixed with 5 mL 4:1 methanol-water in 50 mL centrifuge tube and vortexed at 3000 rpm for 30 s. The mixture was subjected to sonication in ultrasonic water bath (Branson 2510MT Ultrasonic Cleaner, Darmstadt, Germany) with a frequency of 40 Hz at 25 °C for 30 min. The mixture was then centrifuged at 4000 rpm for 15 min to separate the supernatant and precipitates. The supernatant was concentrated using a rotary evaporator (Heidolph Instruments GmbH and Co.KG, Schwabach, Germany) and freeze-dried using Labconco^®^ FreeZone Freeze Drier System (Kansas City, MO, USA) to yield the OPL crude extracts. The extraction process was carried out in triplicates. The steps were repeated using different solvents namely hexane, ethyl acetate, ethyl acetate-methanol and absolute methanol. To aid visualization, Figure 1 showed graphical flow chart of sample preparation of crude extract.

### 2.5. Preparation of Acid Hydrolyzed Oil Palm Leaf (OPLAH) Extract

The acid hydrolysis was performed as described previously Tahir et al. [12], with slight modifications. 10 g of crude OPL extract was added into 100 mL water then 100 mL 12 M concentrated hydrochloric acid was added to the extracts in capped tube. The mixture was incubated in water bath at 85 °C for 2 h. The extract was cooled down to room temperature. 40 mL methanol was added into the mixture. The solution was vortexed at 3000 rpm for 30 sec. The solution was sonicated for 10 min at room temperature and filtered using Whatman No 1 filter paper. The hydrolysed extract was dried using the rotary evaporator (Heidolph Instruments GmbH and Co.KG, Schwabach, Germany). The acid hydrolysed extract was freeze dried using Labconco^®^ FreeZone Freeze Drier System (Kansas City, MO, USA) for complete moisture removal. To aid visualization, Figure 1 showed graphical flow chart of sample preparation of acid hydrolyzed extract.

### 2.6. Preparation of Total Flavonoid and Flavonoids C-Glycoside Enriched Fractions

The glass column (2.5 cm × 46 cm) wet packed with 4.4 g (dry weight) of pre-treated XAD7HP resin. The bed volume (BV) of resin was 200 mL. To prepare 5 mg/mL of final concentration, 750 mg of crude aqueous methanolic OPL was dissolved in 150 mL of deionized water and filtered. The mixture was wisely transferred onto the top of the column. After the adsorption equilibrium was reached, an aliquot 150 mL of 95% ethanol was loaded serially into the column and eluted with a constant flow rate of 0.3 mL/min. The combined fractions were condensed at reduced pressure using a rotary evaporator (Heidolph Instruments GmbH and Co.KG, Schwabach, Germany). The total flavonoid enriched extract after evaporation were lyophilized in a Labconco^®^ FreeZone Freeze Drier System (Kansas City, MO, USA), weighed and analysed by UPLC. The enrichment process was repeated for acid hydrolysed OPL (OPLAH) extract by using 40% ethanol to obtain flavonoid *C*-glycoside enriched extract containing isoorientin, orientin, vitexin and isovitexin. To aid visualization, Figure 1 showed graphical flow chart of sample preparation of enriched fractions.

### 2.7. UHPLC-UV/PDA and LC-MS/MS Analysis

OPL extract was separated using C_18_ reversed-phase Acquity UPLC^®^ BEH 1.7 µm particle size and 2.1 mm i.d. × 100 mm length column from Waters (Wexford, Ireland) on Thermo Scientific Ultimate 3000 with a PDA-3000 photodiode array detector and a thermostat column compartment which was maintained at 25 °C during UHPLC analysis. Gradient elution was performed with water:0.1% formic acid:0.063% ammonium formate (solvent A) and acetonitrile:0.1% formic acid (solvent B). The programme gradient proceeded using following sequence of solvent B percentages: 10% for 0–0.6 min, 10–11% for 0.6–1.0 min, 11–11.3% for 1.0–1.5 min, 11.3% for 1.5–5.5 min, 11.3–11.4% for 5.5–8.0 min, 11.4–11.8% for 8.0–8.2 min, 11.8–12% for 8.2–12.0 min, 12–10% for 12.0–13.0 min and 10% for 13.0–18.0 min. The flow rate was constant at 0.40 mL/min. The UV detector was set at 340 nm. The chromatographic peaks were identified by comparing their respective retention times and UV/PDA spectra with those of reference standards, which were eluted in parallel under the same conditions. All samples were prepared at 5 mg/mL and filtered through a 0.22 µm syringe filter (Sartorius AG, Goettingen, Germany) for UHPLC injection.

After going through the UV/PDA detector, the flow was split to allow only 200 µL/min of eluent into electrospray ionization (ESI) source of MS. The MS analysis was done on Q-Exactive Focus Orbitrap LCMS-MS system. The eluent was monitored by ESI-MS under negative mode scanned from *m/z* 67.9 to 1000. ESI was conducted using a spray voltage of 4.2 kV. High purity nitrogen gas was used as dry gas at a sheath gas flow rate of 40 (arbitrary units) and aux gas flow rate of 8 (arbitrary units). Capillary temperature was set at 320 °C while aux gas heater temperature was set at 0 °C.

### 2.8. Cell Culture Maintenance

3T3 mouse fibroblasts (Cell Line Service, Appelheim, Germany) were maintained in complete Dulbecco’s Modified Eagle Medium (DMEM) high glucose media with 10% Fetal Bovine Serum (FBS) and 5% of penicillin-streptomycin in a humidified 5% CO_2_ incubator at 37 °C. The media was changed every 48 h. Cells at 70–80% confluence was ready for seeding and treatment throughout the experiment. 

### 2.9. The Sample Preparation for Treatment

The crude OPL extracts (hexane, ethyl acetate, ethyl acetate-methanol, absolute methanol and aqueous methanol), acid hydrolysed aqueous methanolic OPL extract, flavonoid enriched fractions (total flavonoid fraction and total flavonoids *C*-glycoside fraction), purified flavonoids *C*-glycosides compounds (orientin, isoorientin, vitexin and isovitexin) and allantoin were prepared by dissolving each sample in cell culture medium and sterilized using syringe filter (0.22 µm). Concentration used in the experiment was based on dry weight of the extract (mg/mL). 

### 2.10. 3-(4,5-Dimethylthiazol-2-yl)-2,5-Diphenyl Tetrazolium Bromide [MTT]) Assay

Cytotoxicity assay was conducted to determine the range of concentrations of extract to be used for scratch assay and further in-vitro analysis. The cytotoxicity assay was performed as described previously by Nur et al. [19], with slight modifications. 3T3 cells were cultured in 96 well plates at density 1 × 10^5^ cells/well with DMEM complete media for 24 h. The medium was replaced with 100 µL of tested sample solution at different concentrations (1.56, 3.13, 6.25, 12.5 and 25 µg/mL) for 48 h. The MTT powder was dissolved in phosphate-buffered saline (PBS) at a concentration of 5 mg/mL. 20 µL of MTT solution was added into each well and plates were incubated at 37 °C for 4 h. The medium was replaced with 100 μL DMSO and the absorbance for each well was measured at 570 nm on Tecan Infinite F200 Pro plate reader (Tecan Group Ltd., Männedorf, Switzerland). Allantoin was used as a positive control drug. Cells without treatment were used as negative control and wells with only media were used as blank. Experiments were performed in triplicates. 

### 2.11. Cell Proliferation Assay

The cell proliferation assay was performed as described previously by Gothai et al. [7], with slight modifications. 3T3 cells were seeded on 96 well plates at a density of 1 × 10^5^ (in 100 µL medium) per well and incubated at 37 °C for 24 h. The medium was replaced with 100 µl sample solutions with different concentrations (1.56, 3.13, 6.25 and 12.5 µg/mL) for 48 h. 10 µL of cell counting kit-8 (CCK-8) was then added into each of the wells and incubated for another 4 h according to the manufacturer’s instructions (Dojindo Lab, Kumamoto, Japan). Tecan Infinite F200 Pro plate reader (Tecan Group Ltd., Männedorf, Switzerland) was used to read absorbance at 450 nm. Graph of absorbance against number of cells was plotted to determine the 3T3 cells proliferation according to the kit manufactures instruction. Allantoin was used as a positive control drug. Cells without treatment were used as negative control and wells with only media were used as blank. Experiments were performed in triplicates.

### 2.12. Scratch Assay

The migration and proliferation of cells was examined using the scratch assay method Gothai et al. [7], and Nur et al. [19], with slight modifications. 3T3 cells were seeded in 24-well plates at a density of 3 × 10^5^ cells/well and allowed to grow for 24 h at 37 °C and 5 % CO_2_. A small linear scratch was created in the confluent monolayer by carefully scraping with sterile P200 pipette tips to ensure uniform size and distance was made for all samples. Cells were rinsed with PBS thrice for complete removal of cellular debris before adding the media with different treatment solutions at different concentrations (1.56, 3.13 and 6.25, µg/mL). The wound closures were monitored and photographed by phase contrast microscopy using ×5 magnification at 0 h. After 24 and 48 h of incubation, the subsequent set of images was analysed using Image-J software and percentage of the closed area was measured and compared to the value obtained before treatment. An increase of the percentage of closed area indicated the migration and proliferation of cells. Allantoin was used as a positive control, while drug and cells without treatment was used as negative control. All scratch assays were performed in six replicates.

### 2.13. Statistical Analysis

Data are given as the mean ± standard deviation (SD) and statistical analyses were performed using Minitab statistical software (Version 16, Minitab Inc, State College, PA, USA), IBM SPSS Statistics software (Version 21, SPSS Inc, Chicago, IL, USA) and InStat V2.02 statistical package (GraphPad Software, San Diego, CA, USA). Results were compared to control and treated groups using t-test. Differences were considered as statistically significant at * *p* < 0.05 versus control group. Meanwhile, comparisons between sample concentrations which are a statistically significance (*p* < 0.05) were performed by Tukey’s test, ANOVA.

## 3. Results

### 3.1. Identification and Confirmation of Flavonoids C-Glycosides in OPL Extracts and Flavonoid Enriched Fractions

**Figure 2:**
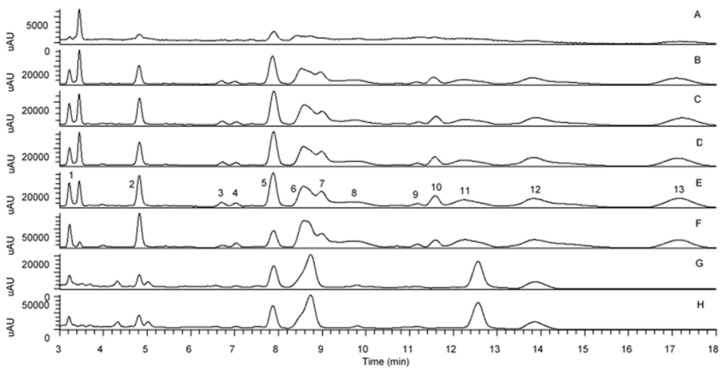
UHPLC-UV/PDA chromatogram of OPL crude extracts and flavonoid enriched fractions (**A**) hexane, (**B**) ethyl acetate, (**C**) ethyl acetate-methanol, (**D**) absolute methanol, (**E**) aqueous methanol, (**F**) total flavonoid enriched fraction, (**G**) acid hydrolysed aqueous methanolic extract and (**H**) total flavonoid *C*-glycoside enriched fraction.

**Figure 3:**
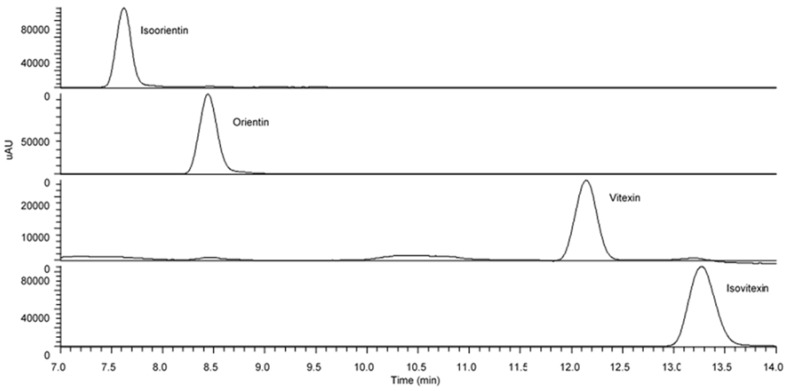
UHPLC-UV/PDA chromatogram of selected reference standards of flavonoid *C*-glycosides (isoorientin, orientin, vitexin and isovitexin).

The UHPLC analysis revealed the pattern of distribution of flavonoid *C*-glycosides presented in oil palm leaves in different solvent extracts and flavonoid enriched fractions. Figure 2 shows the peaks that putatively identified as flavonoid *C*-glycosides in all samples. With the help of the fragmentation patterns from LC-MS/MS data, UV-Vis absorption spectrum and additional supports from previous literature reports [12,13,20], Table 1 lists the 13 identified flavonoid *C*-glycosides, and these peaks were assigned as trace, low, medium, high and very high which derived from their respective peak intensity. The results indicated that the polar solvents managed to extract high concentration of flavonoid *C*-glycosides in comparison with moderate and non-polar solvents including luteolin-6-8-di-*C*-hexose, apigenin-6,8-di-*C*-hexose, apigenin-6-*C*-pentose-8-*C*-hexose, isoorientin, apigenin-6-*C*-hexose-8-*C*-pentose, orientin, luteolin-6-*C*-hexose-8-*C*-deoxyhexose, vitexin, isovitexin and apigenin-6-*C*-hexose-8-*C*-deoxyhexose. Interestingly, flavonoid enriched fraction was found to contain very high concentration of these flavonoids. In order to remove sugar moiety attaching to the aglycone of these flavonoids especially on flavonoid *O*-glycosides, acid treatment was carried out on aqueous methanolic extract which resulting in some of the identified flavonoids were detected as trace or at low concentration. However, four peaks which previously identified as isoorientin, orientin, vitexin and isovitexin were still found moderate intensity even after acid treatment. The acid hydrolysed OPL extract was further fractionated to form flavonoid *C*-glycoside enriched fraction and the content of these four flavonoid *C*-glycosides were found significantly high. These four flavonoid *C*-glycosides which were isoorientin, orientin, vitexin and isovitexin were further confirmed by injecting reference standards (Figure 3). 

### 3.2. Effect of OPL Extracts, Flavonoid Enriched Fractions and Flavonoid C-glycosides on Cell Viability

**Figure 4:**
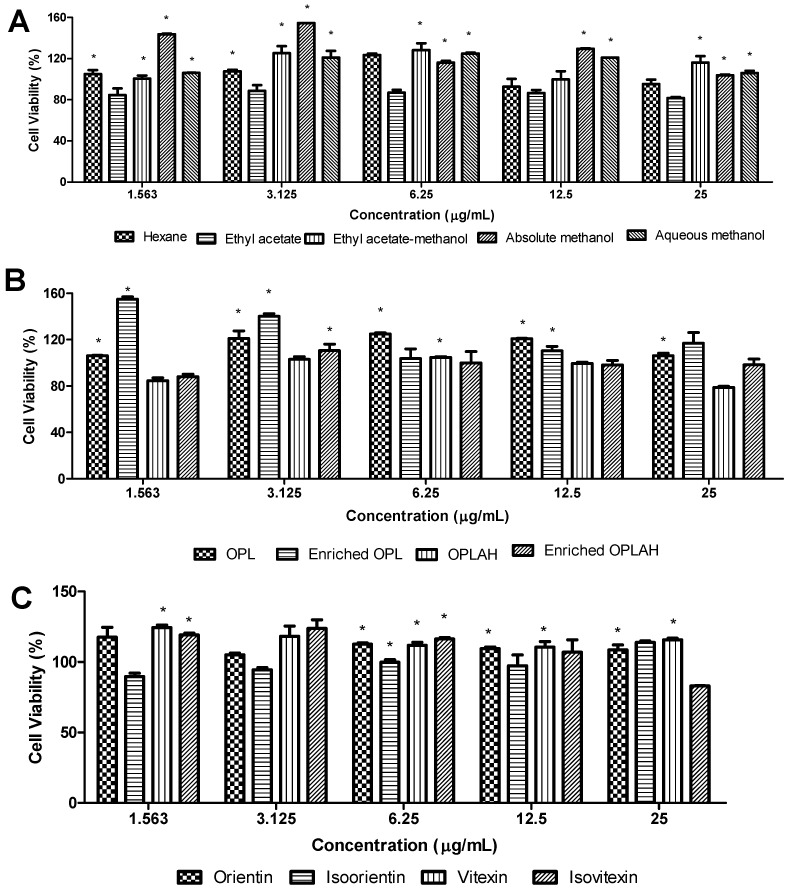
The effects of (**A**) different OPL crude extracts, (**B**) flavonoid enriched fractions and (**C**) flavonoid *C*-glycosides on cell viability of 3T3 fibroblast cells. At 48 h of treatment, the viability of treated cells was evaluated through mitochondrial activity using the MTT assay and calculated by comparing the values from treatment groups with the control group. Values are presented as the mean percentage ± standard deviation (*n* = 3) from three experiments, * *p* < 0.05 versus control group. OPL = aqueous methanolic extract, Enriched OPL = total flavonoid enriched fraction, OPLAH = acid hydrolysed OPL extract, Enriched OPLAH = total flavonoid C-glycoside enriched fraction.

The cytotoxicity effect of OPL extracts, flavonoid enriched fractions and flavonoid *C*-glycosides was determined by MTT assay in 3T3 cells treated with different gradient concentrations (1.56, 3.13, 6.25, 12.5 and 25 µg/mL). Figure 4 shows that all concentrations of OPL extracts, flavonoid enriched fractions and flavonoid *C*-glycosides have no toxicity effect on 3T3 cells indicated by the cell viability percentage reached above 70% [21]. The results showed that the concentration of samples at 1.563–25 µg/mL showed 79–155% cell viability after 48 h incubation. The cytotoxicity test has revealed that the higher the sample concentration, the lower the percentage of cell viability. At a dose of 25 µg/mL and below, OPL crude extracts except hexane and ethyl acetate extracts, have shown more than 100% of cell viability, whereas at the dose of 6.25 µg/mL and below, the flavonoid enriched fractions reached more than 100% of cell viability. Moreover, all flavonoid *C*-glycosides except isoorientin showed more than 100% of cell viability at a dose of 12.5 µg/mL. Although there is a sign of a decrease in percentage cell viability as the concentration increases, the crude extracts, flavonoid enriched fractions and flavonoid *C*-glycosides are considered non-toxic by showing cell viability above 70%. Therefore, we have selected lower concentrations (1.56, 3.13, 6.25 and 12.5 µg/mL) of samples for further wound healing studies. 

### 3.3. Effect of OPL Extracts, Flavonoid Enriched Fractions and Flavonoid C-glycosides on Cell Proliferation

**Figure 5:**
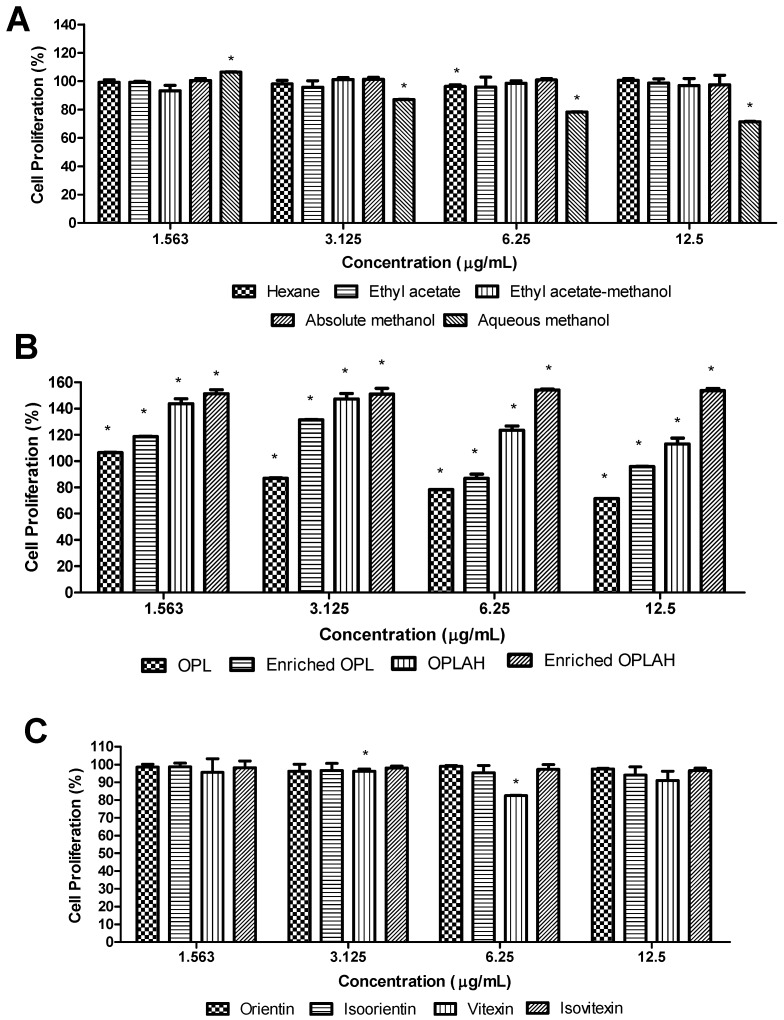
The effects of treatment of (**A**) different OPL crude extracts, (**B**) flavonoid enriched fractions and (**C**) flavonoid *C*-glycosides on cell proliferation of 3T3 fibroblast cells. The proliferation test was estimated by cell counting kit-8 assay and calculated by comparing the values from treatment groups with the control group. Values are presented as the mean percentage ± standard deviation (*n* = 3) from three individual experiments. * *p* < 0.05 versus control group. OPL = aqueous methanolic extract, Enriched OPL = total flavonoid enriched fraction, OPLAH = acid hydrolysed OPL extract, Enriched OPLAH = total flavonoid *C*-glycoside enriched fraction.

The cell proliferation of OPL extracts, flavonoid enriched fractions and flavonoid *C*-glycosides was evaluated by CCK-8 (water-soluble tetrazolium salt, WST-8) assay in 3T3 cells treated with four different gradient concentrations ranging from 1.563 to 12.5 µg/mL, and the results were presented in Figure 5. In general, the samples showed good cell proliferative activity with percentages above 70% at all concentrations. At first, the comparison between crude extracts after being extracted with different polarity of solvents demonstrated that these extracts almost reached 100% cell proliferation rate at different concentrations with no significant difference except for aqueous methanolic extracts. Furthermore, both flavonoid enriched fractions obtained showed better cell proliferation rate in comparison with their respective crude and acid hydrolysed extracts. Interestingly, the highest cell proliferation rate was obtained after 48 h incubation by flavonoid *C*-glycoside enriched fraction with 154.24% at 6.25 µg/mL. The individual flavonoid *C*-glycosides including orientin, isoorientin, vitexin and isovitexin were also tested on their cell proliferative activity resulting in these compounds nearly reached 100% cell proliferation at all tested concentrations with no significant difference. However, the trend consistently exhibited as the concentrations of the samples increased, the cell proliferation rate decreased which could be clearly observed in aqueous methanolic extract and its flavonoid enriched fraction (Figure 5B). The 3T3 cells proliferated better at low concentration (1.563 µg/mL) of aqueous methanolic extract and its flavonoid enriched fraction with 106.47% and 118.60%, respectively. Hence, the results indicated the samples including all crude extracts, flavonoid enriched fractions and flavonoid *C*-glycosides did not affect the cellular activity of 3T3 fibroblast cells within the concentration ranges. 

### 3.4. Effect of OPL Extracts, Flavonoid Enriched Fractions and Flavonoid C-glycosides on Cell Migration and Proliferation Activities

**Figure 6:**
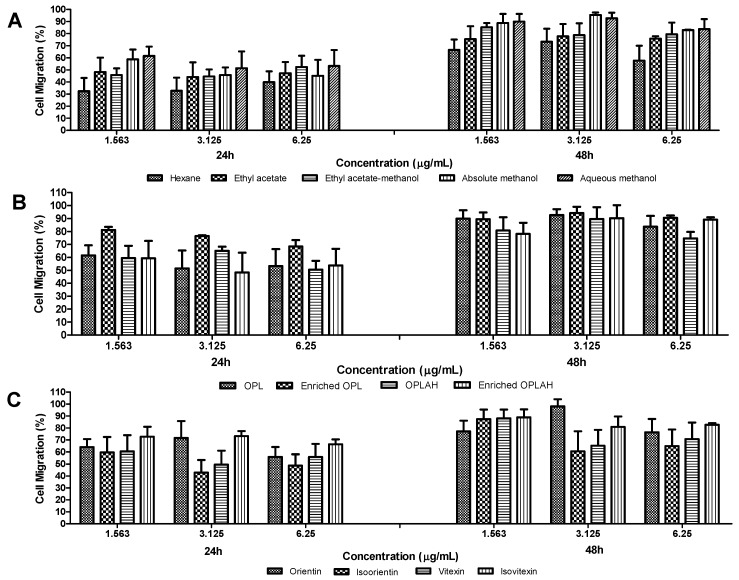
The effects of treatment of (**A**) different OPL crude extracts, (**B**) flavonoid enriched fractions and (**C**) flavonoid *C*-glycosides on cell migration and cell proliferation assay of 3T3 fibroblast cells. The migration and proliferation effects were estimated by percentage of migration and proliferation of cell (%) by comparing the values from 0 h, 24 h and 48 h. Values are presented as the mean percentage ± standard deviation with six replications (*n* = 6). Negative control (24 h; 20.33% ± 6.74, 48 h; 40.35% ± 3.33). Allantoin (24 h; 61.82% ± 9.86, 48 h; 94.96% ± 1.37). OPL = aqueous methanolic extract, Enriched OPL = total flavonoid enriched fraction, OPLAH = hydrolysed aqueous methanolic extract, Enriched OPLAH = total flavonoid *C*-glycoside enriched fraction.

**Figure 7:**
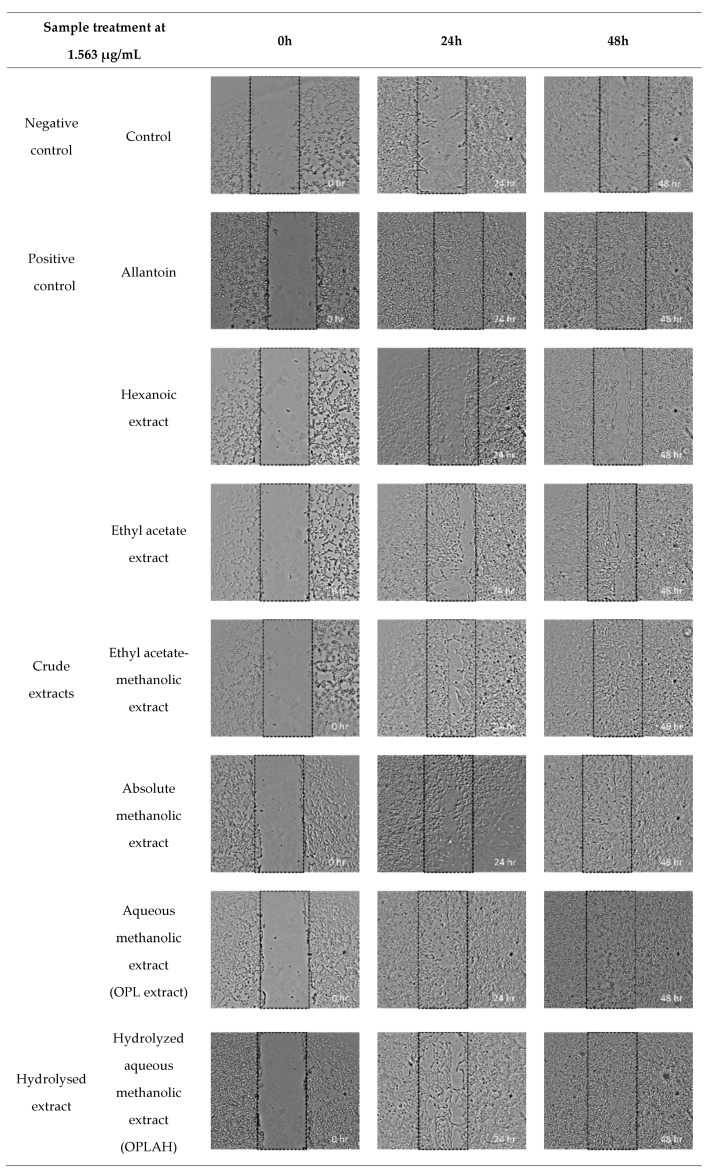

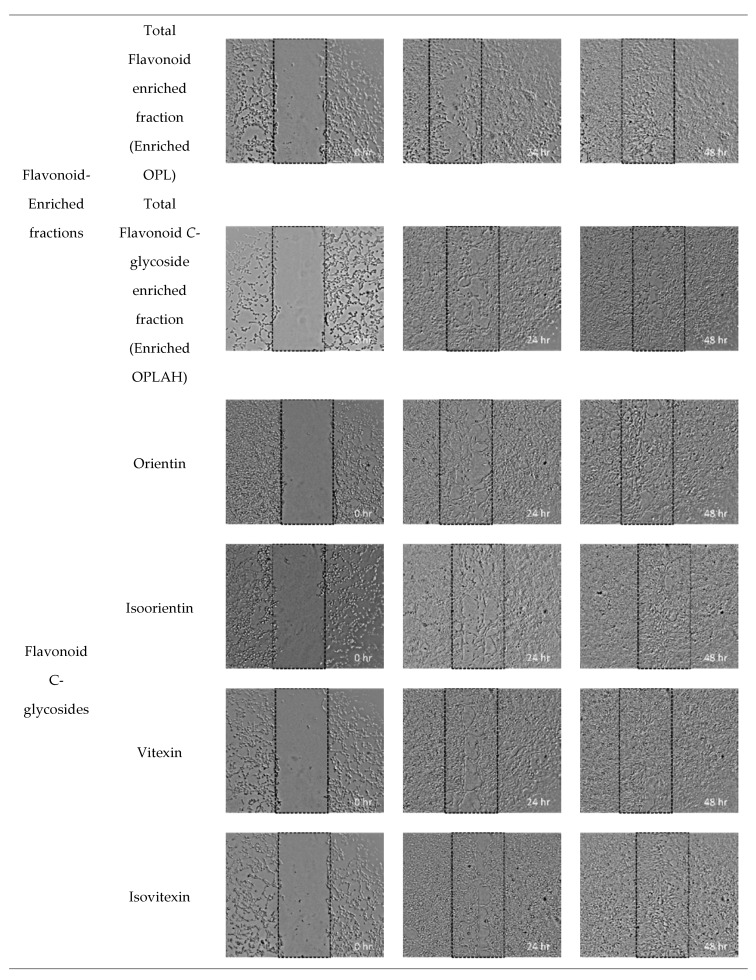
Digital images show the effect of different OPL crude extracts, hydrolysed extract, flavonoid enriched fractions and flavonoid *C*-glycosides on 3T3 fibroblast migration and proliferation *via* scratch assay at 1.563 µg/mL. Migration and proliferation of fibroblast cells were captured and measured using light microscope attached to a digital camera. Magnification (×5).

The migration and proliferation activities of 3T3 fibroblasts were evaluated using a scratch assay which measures the progression of the wound closure on scratch wounded cells and validates the result of the cell proliferation assay. Figure 6 shows the cell migration percentage of OPL extracts, flavonoid enriched fractions and flavonoid *C*-glycosides on 3T3 fibroblasts after 24 h and 48 h treatment, while Figure 7 displays the images of scratch assays on 3T3 cells at 0 h, 24 h and 48 h post injury time without treatment (control) and with treatment at a concentration of 1.563 µg/mL. There was mild migration of cells observed in negative control with 40.35% and a progressive migration and proliferation of cells was noticed in positive control (allantoin) with 94.96% after 48 h incubation. Generally, the results showed enhanced migration and proliferation in all samples after 24 h and the cells in some of these samples migrated and proliferated more than 90% after 48 h of treatment. As shown in cell viability and cell proliferation, a similar trend was observed which showing the cells migrated better at lower concentration than higher concentration in all samples. Among crude extracts, the samples extracted from methanol and its mixtures enhanced the migration and proliferation the cells more rapidly than ethyl acetate and hexane which could be observed as early as 24 h of the treatment. The highest cell migration and proliferation rate was obtained at 3.125 µg/mL by absolute methanolic extract with 95.27% followed by aqueous methanolic extract with 92.56%. Furthermore, the highest activity of the flavonoid enriched fractions in promoting migration was similar with their respective crude and acid hydrolyzed extracts at a concentration of 3.125 µg/mL with 92.56–94.17% and 89.54–90.20%, respectively. Moreover, orientin reached 98.16% at a concentration of 3.125 µg/mL while isoorientin, vitexin and isovitexin reached highest cell migration with 87.40–88.89% at a concentration of 1.563 µg/mL.

## 4. Discussion

The UHPLC-UV/PDA and LC-MS/MS analysis of each extract and flavonoid enriched fractions provides a comprehensive yet putative investigation of flavonoid *C*-glycosides presented in these samples. Four flavonoid *C*-glycosides were confirmed found in the extracts and enriched fractions including isoorientin, orientin, vitexin and isovitexin by comparing with available standards which resulted in peaks appearing on the crude extract and flavonoid enriched fraction chromatograms. Hence, based on the results of cell proliferation and migrations, these four compounds may serve as lead in the breakthrough of therapeutic bioactive agents that may improve wound healing. Generally, these flavonoid *C*-glycosides were reported to possess high antioxidant [22,23,24], and anti-inflammatory activities [16,17], and may be associated with wound healing enhancement. Further studies should be conducted on the correlation of these activities and wound healing properties in OPL extracts and flavonoid enriched extracts. 

Wound healing occurs as cellular feedback to skin damage which involves instigation of fibroblasts, endothelial cells and macrophages [25]. The restoration of structure and function to the injured site is closely associated with fibroblast proliferation. Even though the wound site can be restored naturally by specific repair mechanisms, therapeutic agents that are able to stimulate and promote the cell proliferation and migration would be an added advantage in improving and promoting the wound healing process especially for targeted groups such as elderly and chronically ill people who suffered from a delayed wound healing process. The present study demonstrated the effect of the crude extracts, enriched fractions and flavonoid *C*-glycosides from oil palm (*Elaeis guineensis* Jacq.) mature leaves in enhancing the cell proliferation and migration of 3T3 fibroblast cells. 

The cell viability of the crude extracts, flavonoid enriched fractions and flavonoid *C*-glycosides at five different concentrations were determined to ensure the effect of these sample treatments on fibroblast proliferation and migration were not interfered with by any toxicity. Based on the cytotoxicity results, almost all samples did not cause any cytotoxicity effects on 3T3 fibroblast cells within tested concentrations (1.563–25 µg/mL). However, the cells were more viable at low concentrations than higher concentrations as the number of cells decreased as the concentration increased. The results are in agreement with previous reports that mentioned the *Spirulina platensis* crude extracts were also non-toxic at low concentrations [19], as well as in *Moringa oleifera* crude extracts and fractions [7,23]. 

Both MTT and WST-8 colorimetric assays are able to determine the cell viability in cytotoxicity and cell proliferation studies. In this study, the selection of water-soluble tetrazolium salts, WST-8 assay for the cell proliferation study in comparison with MTT assay is mainly due to the high sensitivity of WST-8 assay to express the numbers of living cells resulted from the amount of the yellow-colour formazan dye, generated by the activities of dehydrogenases in cells since the dehydrogenase activities in cells are directly proportional to the number of living cells. Several studies are in agreement with the high sensitivity shown by WST-8 assay in cell proliferation activity [7,26,27]. 

Proliferation and migration of cells are crucial for wound healing, especially during re-epithelization, as the fast proliferated fibroblasts will provide a sufficient supply of cells to migrate rapidly and cover the wound site [14,28]. From the results, all samples showed good cell proliferative activity within the range of tested concentrations. The wide polarities of compounds presented in aqueous methanolic extracts may contribute to such performance. However, when the extract was hydrolysed with acid and further enriched, the cell proliferation rate was higher than crude samples, indicating acid hydrolysis helped to remove undesired compounds that may reduce the proliferation rate. The individual flavonoid *C*-glycosides that were expected to present in the OPL were also evaluated on their cell proliferation ability, resulting all four compounds; isoorientin, orientin, vitexin and isovitexin were able to proliferate the cells rapidly by showing more than 90% proliferation activity in most of the tested concentrations with no significant difference between these concentrations. 

Furthermore, the samples extracted from methanol and its mixtures showed better migration and proliferation activities than ethyl acetate and hexane, indicating polar solvents managed to extract wide range polarity of compounds that may have wound healing properties. Though both flavonoid enriched fractions showed high migration and proliferation activities, however there were no significant difference with their respective extracts. Unlike cell proliferation activity, total flavonoid enriched fraction showed better migration activity than flavonoid *C*-glycoside enriched fraction, indicating combination of different groups of flavonoids present in total flavonoid enriched extract may contribute to the cell migration and proliferation activities. Again, isoorientin, orientin, vitexin and isovitexin were evaluated on their respective migration ability resulting in isovitexin showed the highest migration and proliferation rate at all concentration followed by orientin, vitexin and isoorientin. However, similar to the cell proliferation results, all the samples constantly showed higher migration and proliferation activity at lower concentration than higher concentration, indicating the crude and flavonoid enriched fractions of OPL may act as anti-proliferative agent at higher concentration due to the accumulation of high amount of flavonoid *C*-glycosides which may be related to the activation of caspases and inducing apoptosis [7,29]. Meanwhile, the positive effect of wound healing at low concentration may serve as signalling messengers in the cells and may aid in the angiogenesis process [30,31]. 

## 5. Conclusions

The study suggested that the mixture of methanolic extracts and flavonoid enriched fractions containing flavonoid *C*-glycosides including isoorientin, orientin, vitexin and isovitexin obtained from oil palm (*Elaeis guineensis* Jacq.) mature leaves were non-toxic below 25 µg/mL and most effective in promoting wound healing process by showing rapid proliferation and migration of 3T3 fibroblast cells. Therefore, the OPL extracts and flavonoid enriched extracts can be an alternative therapeutic wound healing agent. However, there is a need for scientific validation and safety assessment before the extracts or flavonoid enriched fractions from OPL could be commercialized for alternative wound healing remedy.

## Figures and Tables

**Table 1:** Identification of flavonoid *C*-glycosides present in oil palm mature leaf crude extracts and flavonoid enriched fractions by LC-MS and UHPLC-UV/PDA.

Peak	t_R_ (min)	λ_max_, (nm)	[M−H]^−^ (*m/z*)	Formula	Key MS/MS Fragments (*m/z*)	Compound	He	Ea	EM	Abs. Me	Aq. Me	Enriched OPL	OPLAH	Enriched OPLAH
1	3.17	272, 348	609.1411	C_27_H_30_O_16_	519.1104 489.0998, 429.0786, 399.0696, 369.0585	Luteolin-6-8-di-*C*-hexose	+	++	++	++	+++	++++	+	++
2	4.88	272, 336	593.1464	C_27_H_30_O_15_	503.1155, 473.1051, 383.0739, 353.0638	Apigenin-6,8-di-*C*-hexose	+	++	++	++	+++	++++	+	++
3	6.72	272, 346	609.1411	C_27_H_30_O_16_	489.1001, 429.0789, 399.0679, 369.0604	Luteolin-6-8-di-*C*-hexose	-	+	++	++	+++	++++	-	-
4	7.10	272, 334	563.1359	C_26_H_28_O_14_	473.1053, 443.0949, 383.0742, 353.0639	Apigenin-6-*C*-pentose-8-*C*-hexose	-	+	++	++	+++	++++	-	-
5	7.86	270, 348	447.0896	C_21_H_20_O_11_	357.0588, 339.0480, 327.0483, 297.0379, 285.0381	Isoorientin (Luteolin-6-*C*-hexose)	+	+	++	++	+++	++++	++	++++
6	8.52	272, 336	563.1359	C_26_H_28_O_14_	473.1051, 443.0949, 383.0741, 353.0638	Apigenin-6-*C*-hexose-8-*C*-pentose	+	++	+	++	+++	++++	+	+
7	9.00	270, 350	447.0896	C_21_H_20_O_11_	357.0587, 339.0476, 327.0485, 297.0378, 285.0380	Orientin (Luteolin-8-*C*-hexose)	-	+	++	++	+++	++++	+	++++
8	9.87	270, 348	593.1464	C_27_H_30_O_15_	473.1049, 429.0792, 369.0590, 357.0589, 327.0485	Luteolin-6-*C*-hexose-8-*C*-deoxyhexose	-	+	+	++	+++	++++	-	-
9	11.22	274, 334	563.1359	C_26_H_28_O_14_	503.1168, 473.1056, 443.0950, 383.0743, 353.0639	Apigenin-6-*C*-pentose-8-*C*-hexose	-	+	+	+	++	++++	-	-
10	11.60	272, 336	593.1464	C_27_H_30_O_15_	473.1067, 413.0846, 369.0590, 357.0589, 293.0434	Luteolin-6-*C*-hexose-8-*C*-deoxyhexose	-	+	+	++	+++	++++	-	-
11	12.44	270, 338	431.0947	C_21_H_20_O_10_	341.0639, 323.0529, 311.0536, 283.0589	Vitexin (Apigenin-6-*C*-hexose)	-	+	+	++	+++	++++	++	++++
12	13.85	270, 338	431.0947	C_21_H_20_O_10_	341.0638, 323.0536, 311.0536, 283.0588	Isovitexin (Apigenin-8-*C*-hexose)	-	+	++	++	+++	++++	++	++++
13	17.19	270, 338	577.1306	C_27_H_30_O_14_	457.1098, 413.0845, 353.0630, 341.0640, 311.0536, 293.0432	Apigenin-6-*C*-hexose-8-*C*-deoxyhexose	-	+	+	++	+++	++++	-	-

He = hexane, Ea = ethyl acetate, EM = ethyl acetate-methanol, Abs. Me = absolute methanol, Aq. Me = aqueous methanol, Enriched OPL = total flavonoid enriched fraction, OPLAH = acid hydrolysed OPL extract, Enriched OPLAH = total flavonoid *C*-glycoside enriched fraction, (-) = trace, (+) = low concentration, (++) = medium concentration, (+++) = high concentration and (++++) = significantly high concentration.
